# High-resolution chest CT using 1024-matrix reconstruction: phantom and clinical evaluation of image quality and post-processing capability

**DOI:** 10.1007/s11604-026-01964-0

**Published:** 2026-03-06

**Authors:** Kana Hayashi, Yoshiyuki Ozawa, Hirotsugu Ohkubo, Katsuhiro Okuda, Hiroshi Kunitomo, Seita Watanabe, Katsuya Kato, Akio Hiwatashi

**Affiliations:** 1 Department of Radiology, Nagoya City University West Medical Center, Nagoya, Japan; 2 Department of Diagnostic Radiology, Fujita Health University School of Medicine, 1-98, Dengakugakubo, Kutsukake-cho, Toyoake, Aichi 470-1192 Japan; 3 Department of Respiratory Medicine, Allergy and Clinical Immunology, Nagoya City University Graduate School of Medical Sciences, Nagoya, Japan; 4 Department of Thoracic and Pediatric surgery, Graduate School of Medical Sciences, Nagoya City University, Nagoya, Japan; 5 Department of Medical Equipment Engineering, Fujita Health University School of Medical Sciences, Toyoake, Japan; 6 Department of Radiology, Nagoya City University Hospital, Nagoya, Japan; 7 Department of Radiology, Nagoya City University Graduate School of Medical Sciences, Nagoya, Japan

**Keywords:** Multidetector Computed Tomography, Thorax, Phantoms, Imaging, Lung diseases, Interstitial

## Abstract

**Purpose:**

To evaluate the image quality and clinical utility of high-resolution computed tomography (CT) images reconstructed using post-processing with a 1024 × 1024 matrix.

**Materials and methods:**

Four CT image types were reconstructed: type A (512 × 512 matrix, 1-mm slice thickness), type B (1024 × 1024 matrix, 1-mm slice thickness), type C (1024 × 1024 matrix, 0.5-mm slice thickness), and type D (retargeted zoomed images; 512 × 512 matrix, 1-mm slice thickness). Type D images served as the reference standard with a field of view (FOV) half that of the other image types. Phantom image quality was objectively assessed using the task transfer function (TTF), noise power spectrum (NPS), and attenuation profile analysis. In the clinical study, two radiologists independently evaluated image quality in 93 patients, including abnormal lung findings, normal structures, and subjective image noise.

**Results:**

In the phantom study, post-processing 1024 × 1024 matrix reconstruction derived from the same sinogram as conventional 512 × 512 reconstruction improved spatial resolution while maintaining comparable noise characteristics at the same slice thickness. Clinically, image quality of normal structures and abnormal lesions was significantly higher in the order of type C > type B > type A (*p* < 0.0001). Subjective image noise followed the same order. Image quality and noise levels of type B images were comparable to those of type D images.

**Conclusion:**

Post-processing 1024 × 1024 matrix reconstruction improves spatial resolution and provides clinically useful high-resolution CT images comparable to retargeted zoomed reconstruction without the limitation of a reduced FOV.

**Supplementary Information:**

The online version contains supplementary material available at 10.1007/s11604-026-01964-0.

## Introduction

High-resolution computed tomography (CT) is widely used for evaluating various lung diseases, especially diffuse lung diseases [1, 2], and the matrix size is one of the most important factors for obtaining high-resolution images [1, 3]. When using a standard matrix size of 512 × 512 pixels, retargeted zoomed images can be used to create high-resolution images with smaller pixel sizes and allow greater depiction of the subtle details of normal lung structures and abnormal CT findings [1, 3]. However, retargeted zoomed images have a limited field-of-view (FOV) and cannot show the whole lung. Currently, larger matrix sizes of 1024 × 1024 or 2048 × 2048 pixels are available in ultra-high-resolution CT systems equipped with dedicated high-resolution hardware, which can overcome the limitations of small field-of-view associated with retargeted zoomed CT images [4–12]. In contrast, although the CT system used in this study can reconstruct images with a 1024 × 1024 matrix, this capability is achieved through post-processing image reconstruction on a conventional detector system, rather than through hardware-based ultra-high-resolution CT. Therefore, true ultra-high-resolution CT remains dependent on dedicated scanner hardware. In addition to such hardware-based methods, a recently developed post-processing reconstruction method can achieve high-resolution CT images with a 1024 × 1024 matrix size, and this method is not limited to certain CT scanners [13, 14]. This post-processing (precision matrix) reconstruction method makes it possible to reconstruct images with various matrix sizes that are greater than the standard matrix size of 512 × 512, including matrix sizes of 768 × 768 and 1024 × 1024, from the same sinogram. This method also has the advantage of allowing the assessment of both lungs simultaneously without requiring retargeted zoomed reconstruction of each lung. However, to the best of our knowledge, few studies have examined such post-processing reconstruction [13, 14].

Therefore, the purpose of this study was to evaluate the quality of high-resolution chest computed tomography (CT) images reconstructed via post-processing with a 1024 matrix size based on sinograms derived from images with a 512 matrix size.

## Materials and methods

### Subjects

For the phantom study, we used the CTP528 module of the Catphan504 (The Phantom Laboratory, NY, USA) as a water phantom of 200 mm in diameter, in which an acrylic cylinder measuring 50 × 50 mm was placed, as shown in Supplement Figure S1.

For the clinical study, consecutive patients who underwent chest CT with 1024 × 1024 matrix reconstruction between June 2021 and July 2021 were included in this study. This study was approved by the institutional review board of Nagoya City University Hospital. The need to obtain written informed consent from the patients was waived because this was a retrospective study.

### CT image acquisition and reconstruction

**Fig. 1:**
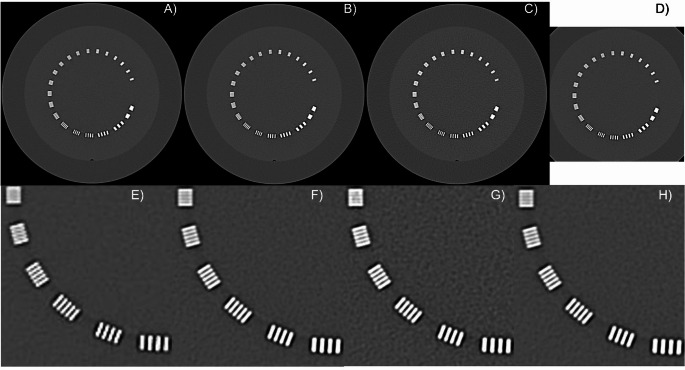
CT images of the phantom. **A**–**D** Four types of CT images reconstructed using the following settings: pA) the lung setting with a 512 × 512 matrix and 1-mm slice thickness and interval; pB) the lung setting with a 1024 × 1024 and 1-mm slice thickness and interval; pC) the lung setting with a 1024 × 1024 matrix and 0.5-mm slice thickness and interval; and pD) the retargeted zoomed lung setting with a 512 × 512 matrix and 1-mm slice thickness and interval, respectively. E-H) Magnified CT images corresponding to pA)–pD), respectively. The results of the objective image evaluation of this phantom were described in the Results section

Details of the phantom study are provided in the Supplementary Materials. A 192-slice dual-source CT scanner (Somatom Force, Siemens Healthineers, Erlangen Germany) was used. The 4 types of reconstructed images were produced using the following settings: pA) the lung setting with a 512 × 512 matrix and 1-mm slice thickness and interval values, pB) the lung setting with a 1024 × 1024 and 1-mm slice thickness and interval values, pC) the lung setting with a 1024 × 1024 matrix and 0.5-mm slice thickness and interval values, and pD) the retargeted zoomed lung setting with a 512 × 512 matrix and 1-mm slice thickness and interval values (Fig. 1).

For the clinical study, all patients underwent whole-thorax CT scans during the full-inspiration phase, using the same scanner as was used in the phantom study. The scan parameters were as follows: collimation, 0.6 mm × 192 rows; tube voltage, 120 kVp; quality reference mAs (mean ± standard deviation (SD)), 161 ± 45; pitch, 0.9; gantry rotation time, 0.5 s; CTDIvol (mean ± SD), 10.8 ± 3.0 mGy; dose-length product (DLP) (mean ± SD), 419 ± 125 mGycm. The original scan data were then reconstructed to produce 4 types of images. Axial CT images were reconstructed using the Bl57 kernel and an iterative reconstruction of ADMIRE strength of 3 for the lung setting. The size of the FOV (mean ± SD) was 310 ± 30 mm. Retargeted zoomed images of the right lung were also reconstructed with a FOV of half of this size; i.e., the size of the zoomed FOV (mean ± SD) was 155 ± 15 mm. In addition, 1024 × 1024 matrix images were obtained using the Precision Matrix. The 4 types of reconstructed images were produced using the following settings: cA) the lung setting with a 512 × 512 matrix and 1-mm slice thickness and interval values, cB) the lung setting with a 1024 × 1024 matrix and 1-mm slice thickness and interval values, cC) the lung setting with a 1024 × 1024 matrix and 0.5-mm slice thickness and interval values, and cD) the retargeted zoomed lung setting with a 512 × 512 matrix and 1-mm slice thickness and interval values. The cA images were standard thin-slice CT images, and the cD images were conventional high-resolution CT images.

### Phantom study

The resolution and noise characteristics of the CT images were evaluated using the task transfer function (TTF) [15–17] and noise power spectrum (NPS) [18, 19], respectively. We also obtained the attenuation profile of each CT image using a 5-line pairs/cm object of the CTP528, which is a high-contrast module of Catphan 504, and measured the curves using the ImageJ (version 1.53 m) software (NIH, Bethesda, MD). Further details of the phantom study are described in the Supplementary Materials [15–22].

### Clinical study

#### Subjective image analyses

An 8.3-megapixel, 23.8-inch color liquid crystal display monitor was used for the image analyses. The images were displayed with a window level/width of -550/1500 Hounsfield units (HU) for the lung setting and a window level/width of 300/3000 HU for the bone setting. Two radiologists without knowledge of the image type independently evaluated the following CT features: normal structures (the 4th and 5th bronchi in the right lower lobe, the 4th and 5th pulmonary vessels in the right lower lobe, and the vertebral cortex and trabeculae of the same level of the bronchi and vessels evaluated), abnormal CT findings (emphysema, ground-glass opacity, consolidation, reticular opacities, honeycombing, and centrilobular nodules), and subjective overall image noise. The abnormal CT findings on image cA were pointed out and recorded by reader 1 (Y.O.), in advance, and scoring of the abnormal CT findings was performed according to the record. The two radiologists (reader 1, Y.O. with 19 years of experience; reader 2, K.H. with 10 years of experience) compared the cA, cB, and cC images with the cD images by using the following scoring systems. The four images (cA, cB, cC, and cD) were displayed simultaneously on the monitor, with cA, cB, and cC randomly positioned on the screen. For normal anatomical structures and pulmonary lesions, ‘1’ indicated poor image quality (i.e., it was possible to detect structures but difficult to clearly evaluate their margins or internal characteristics); ‘2’ indicated fair image quality (i.e., the margins or internal characteristics could be detected and evaluated as well as in the reference images); ‘3’ indicated excellent image quality (i.e., it was easy to detect findings and to evaluate their margins or internal characteristics without any indistinct findings). For image noise, ‘1’ indicated strong presence and non-diagnostic; ‘2’ indicated strong presence but diagnostic; ‘3’ indicated moderate presence (i.e., similar to those in the reference images); ‘4’ indicated slight presence or almost absent. For each abnormal CT finding, only the image quality of three predetermined abnormal findings was evaluated when four or more abnormalities of the same type were present in the lung.

#### Objective image analyses

The objective image noise of each image was evaluated by calculating standard deviation (SD) values for circular regions of interest (ROI) of 10 mm in diameter. According to previous studies [4, 5], we adjusted the noise measurement method in the present study. Because all images were displayed with a similarly limited area to maintain blinding, the available air space for noise measurement was restricted. Therefore, image noise was assessed using two homogeneous air spaces anterior to the right chest wall, with ROIs placed at identical locations on each selected image (the cA, cB, and cC images), except for the retargeted zoomed images (the cD images). The average SD values for two ROIs were used for comparisons among the CT images.

#### Statistical analyses

In the clinical study, the Friedman test and Wilcoxon signed rank test with Bonferroni’s correction were used for the subjective and objective analyses, respectively. The weighted kappa test was used to test inter-rater reliability with the following predefined levels of agreement: 0–0.20, poor; 0.21–0.40, fair; 0.41–0.60, moderate; 0.61–0.80, substantial; and 0.81–1.00, almost perfect agreement. *P*-values of < 0.05 (with the Bonferroni correction < 0.017) were considered significant. The weighted kappa test and Wilcoxon signed rank test were performed with STATA/SE 17.0 (StataCorp, TX, USA), and the Friedman test was performed with EZR version 1.27 (Saitama Medical Center, Jichi Medical University, Saitama, Japan), which is a graphical user interface for R (The R Foundation for Statistical Computing, Vienna, Austria).

## Results

### Phantom study

The results regarding image resolution characteristics are shown in Supplement Figure S2. In the lung setting, the peaks of the TTF curves for all image types were observed at a spatial frequency of 0.3 mm^−1^, and the mean TTF values of the pA, pB, pC, and pD images were 1.78, 1.87, 1.86, and 1.89, respectively. Compared with that of the pA images, the TTF values of the pB and pC images showed a 5% improvement, and that of the pD images showed a 7% improvement.

The results regarding noise characteristics are shown in Supplement Figure S3. At a spatial frequency of 0.5 mm^−1^, the mean NPS values of the pA, pB, pC, and pD CT images were 1580, 2126, 6235, and 2010, respectively. CT images with a 1-mm slice thickness (the pB and pD images) showed similar noise characteristics, whereas the pA) images also demonstrated comparable noise characteristics, with approximately a 25% improvement at 0.5 mm^−1^. In contrast, CT images with a 1024 matrix and 0.5-mm slice thickness (the pC images) showed degradation in granularity, and the NPS value of the pC images was 2.93 times as high as that of the images produced with a 1-mm slice thickness and 1024 matrix size (the pB images) at a spatial frequency of 0.5 mm^−1^.

Supplement Figure S4 shows the attenuation profile of the CTP528 bar pattern of each image type. The shape of the profiles in image pA was distorted and its peaks were lower than those for the other image types. The mean contrast values of the pA, pB, pC, and pD images were 2945, 3936, 3983, and 3937, respectively. The contrast values of the pB, pC, and pD images were 1.3, 1.4, and 1.3 times as high as that of the pA images, respectively.

### Clinical study

#### Patient characteristics

Ninety-four patients were enrolled in this study. One patient was excluded because of failure during the image reconstruction. Thus, 93 patients (mean age: 71 years, range: 34–90 years; 41 females and 52 males) were finally included in this study. The evaluated pulmonary lesions were 100 emphysemas, 136 ground-glass opacities, 57 consolidations, 75 reticular opacities, 35 honeycomb lesions, and 73 centrilobular nodules.

#### Subjective image analyses

**Fig. 2:**
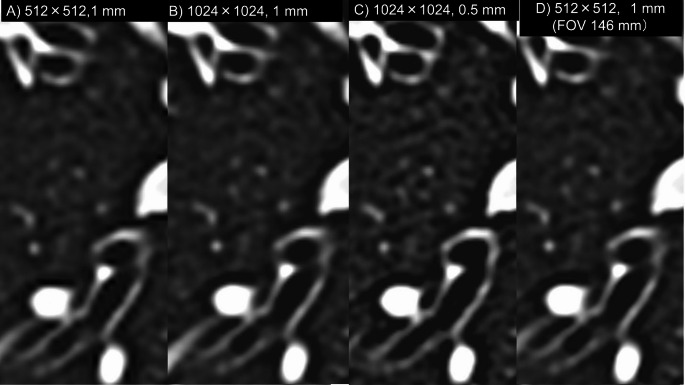
Image quality of normal structures. **A** The lung setting with a 512 × 512 matrix and 1-mm slice thickness and interval, **B** the lung setting with a 1024 × 1024 matrix and 1-mm slice thickness and interval, **C** the lung setting with a 1024 × 1024 matrix and 0.5-mm slice thickness and interval, and **D** the retargeted zoomed lung setting with a 512 × 512 matrix and 1-mm slice thickness and interval. Image A-C are reconstructed with an FOV of 292 mm, whereas image D was reconstructed with an FOV of 146 mm. The clarity of the depiction of normal structures exhibits the following pattern: C > B > A images. The edges of the bronchi and vessels in image A are less clear and smooth compared to those in the other images. The subjective degree of image noise exhibits the following pattern: C > B > A images

**Fig. 3:**
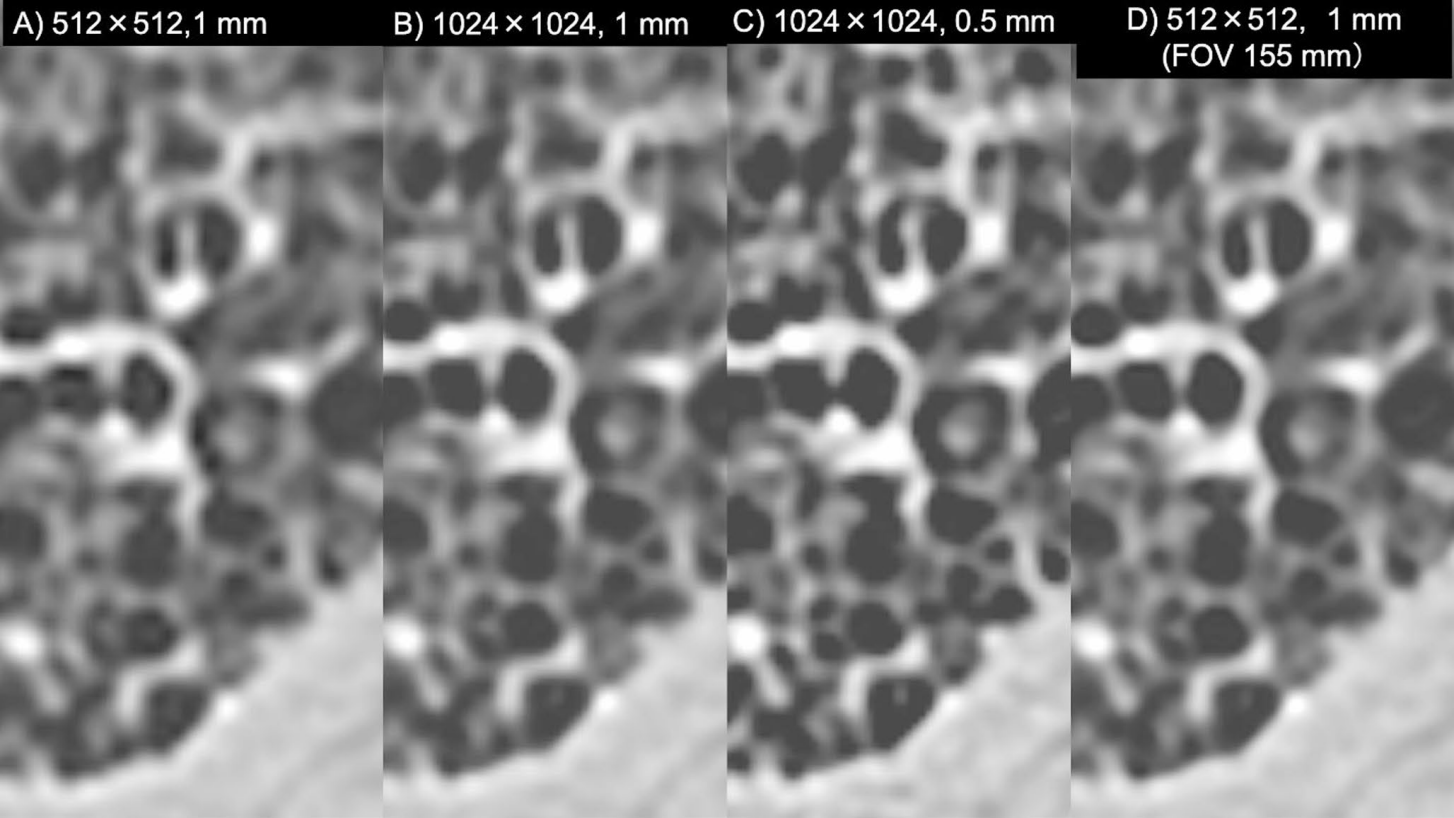
Image quality of abnormal lesions. **A** The lung setting with a 512 × 512 matrix and 1-mm slice thickness and interval, **B** the lung setting with a 1024 × 1024 matrix and 1-mm slice thickness and interval, **C** the lung setting with a 1024 × 1024 matrix and 0.5-mm slice thickness and interval, and **D** the retargeted zoomed lung setting with a 512 × 512 matrix and 1-mm slice thickness and interval. Image A–C are reconstructed with an FOV of 309 mm, whereas image D is reconstructed with an FOV of 155 mm. 83 years-old man with interstitial pneumonia. Both readers found that the image quality of abnormal lesions (reticular lesions, and honeycomb changes) is significantly better in the following order: C > B > A images. Images B, C, and D depict the reticular lesions and honeycomb changes more clearly than image A

The results of the subjective analyses by readers 1 and 2 are shown in Table 1. For both readers, the image quality scores for normal structures (bronchi, pulmonary vessels, the vertebral cortex, and vertebral trabeculae) were significantly higher in the following order: cC > cB > cA images (*p* < 0.0001, respectively) (Fig. 2). Regarding the image quality scores for abnormal CT findings, reader 1 found that they were significantly higher among the image types in the following order: cC > cB > cA images (*p* < 0.0001, respectively), except for in the comparisons of emphysema between the cA and cC images (*p* = 0.14) and in the comparisons of ground-glass opacity between the cB and cC images (*p* = 0.78). On the other hand, reader 2 found that the image quality scores for consolidation, reticular opacities, and honeycombing were significantly higher among the image types in the following order: cC > cB > cA images (*p* < 0.0001, respectively) (Fig. 3). The cB and cC images showed significantly higher scores than the cA images for emphysema and centrilobular nodules (*p* < 0.0001 or *p* = 0.0001, respectively). The cB images showed significantly higher scores than the cA and cC images for ground-glass opacity (*p* < 0.0001). Both readers found that the overall image noise scores were significantly higher in the following order: cA > cB > cC images (*p* < 0.0001, respectively), indicating that the actual image noise was significantly higher in the following order: cC > cB > cA images. The cB images had almost the same image quality as the reference standard cD images.

**Table 1:** Subjective analyses of image quality of the clinical images

	Score (Mean ± SD)			*P* value		
	(cA) 512, 1 mm	(cB) 1024, 1 mm	(cC) 1024, 0.5 mm	(cA) vs. (cB)	(cA) vs. (cC)	(cB) vs. (cC)
Reader 1						
Normal structures						
4th bronchi	1.01 ± 0.10	2.00 ± 0.15	2.72 ± 0.45	< 0.0001*	< 0.0001*	< 0.0001*
5th bronchi	1.02 ± 0.15	2.00 ± 0.00	2.65 ± 0.48	< 0.0001*	< 0.0001*	< 0.0001*
4th pulmonary vessels	1.03 ± 0.18	2.00 ± 0.00	2.35 ± 0.48	< 0.0001*	< 0.0001*	< 0.0001*
5th pulmonary vessels	1.02 ± 0.15	2.00 ± 0.00	2.57 ± 0.50	< 0.0001*	< 0.0001*	< 0.0001*
Cortex of the vertebra	1.15 ± 0.36	2.00 ± 0.00	2.26 ± 0.44	< 0.0001*	< 0.0001*	< 0.0001*
Trabecula of the vertebra	1.11 ± 0.31	2.00 ± 0.00	2.66 ± 0.48	< 0.0001*	< 0.0001*	< 0.0001*
Abnormal CT findings						
Emphysema	1.57 ± 0.50	2.00 ± 0.00	1.69 ± 0.51	< 0.0001*	0.14	< 0.0001*
Ground-glass opacity	1.51 ± 0.50	1.99 ± 0.09	1.99 ± 0.56	< 0.0001*	< 0.0001*	0.78
Consolidation	1.37 ± 0.49	2.00 ± 0.00	2.40 ± 0.49	< 0.0001*	< 0.0001*	< 0.0001*
Reticular opacities	1.03 ± 0.16	2.00 ± 0.00	2.76 ± 0.46	< 0.0001*	< 0.0001*	< 0.0001*
Honeycombing	1.00 ± 0.00	2.00 ± 0.00	2.74 ± 0.44	< 0.0001*	< 0.0001*	< 0.0001*
Centrilobular nodules	1.26 ± 0.47	1.99 ± 0.12	2.40 ± 0.55	< 0.0001*	< 0.0001*	< 0.0001*
Overall image noise	3.22 ± 0.51	2.99 ± 0.10	2.01 ± 0.10	< 0.0001*	< 0.0001*	< 0.0001*
Reader 2						
Normal structures						
4th bronchi	1.01 ± 0.10	2.00 ± 0.00	2.98 ± 0.15	< 0.0001*	< 0.0001*	< 0.0001*
5th bronchi	1.01 ± 0.10	2.00 ± 0.00	2.98 ± 0.15	< 0.0001*	< 0.0001*	< 0.0001*
4th pulmonary vessels	1.00 ± 0.00	1.99 ± 0.10	2.96 ± 0.20	< 0.0001*	< 0.0001*	< 0.0001*
5th pulmonary vessels	1.00 ± 0.00	1.99 ± 0.10	2.98 ± 0.20	< 0.0001*	< 0.0001*	< 0.0001*
Cortex of the vertebra	1.04 ± 0.20	2.00 ± 0.00	2.91 ± 0.28	< 0.0001*	< 0.0001*	< 0.0001*
Trabecula of the vertebra	1.02 ± 0.15	2.00 ± 0.00	3.00 ± 0.00	< 0.0001*	< 0.0001*	< 0.0001*
Abnormal CT findings						
Emphysema	1.25 ± 0.54	2.00 ± 0.00	1.80 ± 0.95	< 0.0001*	0.0001*	0.04
Ground-glass opacity	1.71 ± 0.71	2.00 ± 0.00	1.52 ± 0.78	< 0.0001*	0.05	< 0.0001*
Consolidation	1.11 ± 0.31	2.02 ± 0.13	2.96 ± 0.26	< 0.0001*	< 0.0001*	< 0.0001*
Reticular opacities	1.05 ± 0.23	2.00 ± 0.00	2.99 ± 0.12	< 0.0001*	< 0.0001*	< 0.0001*
Honeycombing	1.06 ± 0.24	2.00 ± 0.00	3.00 ± 0.00	< 0.0001*	< 0.0001*	< 0.0001*
Centrilobular nodules	1.21 ± 0.50	2.00 ± 0.00	2.00 ± 0.94	< 0.0001*	< 0.0001*	1.00
Overall image noise	3.97 ± 0.18	3.00 ± 0.00	2.00 ± 0.00	< 0.0001*	< 0.0001*	< 0.0001*

SD, standard deviation

*Showed significant difference with Bonferroni correction (*p* < 0.017)

The weighted kappa coefficient, which was used to indicate the degree of inter-rater agreement between the two radiologists, for all subjective evaluations was 0.684 (*p* < 0.0001), indicating substantial agreement.

#### Objective image analyses

The mean and SD image noise values for the cA, cB, and cC images obtained by reader 1 were 22.8 ± 4.5, 25.2 ± 4.0, and 41.3 ± 6.0, respectively. The mean and SD image noise values for the cA, cB, and cC images obtained by reader 2 were 22.4 ± 4.0, 25.0 ± 4.8, and 41.4 ± 8.0, respectively. For both readers, there were significant differences in each comparison (*p* < 0.0001 for cA versus cB images, cB versus cC images, and cA versus cC images).

## Discussion

We investigated whether the high-resolution CT reconstruction method can be used to reconstruct images with matrix sizes of 1024 × 1024, from the same sinograms that are used to produce images with 512 × 512 matrices. In our phantom study, we evaluated the objective image quality of post-processing 1024 × 1024 matrix reconstruction based on the same sinogram as was used for routine 512 × 512 matrix reconstruction. Regarding the TTF in the lung setting, the images with 1024 × 1024 matrix (the pB and pC images) and those produced with the retargeted zoomed lung setting and a 512 × 512 matrix (the pD images) exhibited improved resolution characteristics compared with the images with a 512 × 512 matrix (the pA images). The noise characteristics of the pB and pD images, which had the same slice thickness, but were reconstructed with a 1024 × 1024 matrix and as retargeted zoomed images with a 512 × 512 matrix, respectively, were similar. The attenuation profiles of the CTP528 bar pattern for each image type also indicated that the precision matrix was able to reconstruct high-resolution CT images with a 1024 × 1024 matrix, which is greater than the standard matrix size of 512 × 512, from the same sinogram as was used to reconstruct 512 × 512 matrix images.

In the clinical study, we evaluated the image quality of the following 3 types of CT images: standard thin-slice CT (cA images) and high-resolution chest CT images reconstructed by post-processing 1024 matrix (Precision Matrix) reconstruction with a slice thickness of 1 mm (the cB images) or 0.5 mm (the cC images). Retargeted zoomed images with a 512 × 512 matrix (the cD images) were used as the reference standard. The cB images had almost the same image quality as the reference standard (the cD images). The image quality of normal thoracic structures was significantly better in the following order: the cC > cB > cA images. Both readers found that the image quality of abnormal lesions (specifically consolidation, reticular lesions, and honeycomb changes) was also significantly better in the following order: cC > cB > cA.

The in-plane pixel size is determined by the displayed FOV and the size of the reconstruction matrix. In this clinical study, the mean FOV size was 310 mm for the cA, cB, and cC images, and the mean FOV size of the retargeted zoomed images (the cD images) was 155 mm. Therefore, the mean pixel sizes of the cA, cB, cC, and cD images were 0.6 mm, 0.3 mm, 0.3 mm, and 0.3 mm, respectively. The retargeted zoomed images reconstructed with a FOV that was half the size of the FOVs of the other images had similar spatial resolution to the 1024 × 1024 matrix images, but their FOV was too small to cover both lungs simultaneously, which is sometimes important, e.g., when evaluating the distribution and asymmetry of abnormal findings in diffuse lung diseases [23–25]. It also requires additional time and effort for operators to generate retargeted zoomed images, which reduces the overall efficiency of CT examinations. It is also difficult to evaluate high-resolution images of large patients with a small FOV. Therefore, post-processing 1024 × 1024 matrix reconstruction can overcome such weaknesses.

The clinical utility of high-resolution CT imaging has been reported. The percentage low attenuation volume (%LAV) of the entire lung is used for quantitative analysis of the severity of emphysema in patients with chronic obstructive pulmonary disease. It has been reported that compared with conventional thin-slice CT reconstruction using a 512 matrix and a 0.5-mm slice thickness, high-resolution CT reconstruction of the entire lung using a 1024 × 1024 matrix and a 0.25-mm slice thickness improved the correlation between the %LAV and airflow limitations [7]. For pulmonary nodules, high-resolution CT with 1024 × 1024 matrix reconstruction has been shown to improve qualitative assessment of nodule morphology more consistently than detection or classification in itself. It has been reported that 1024 × 1024 matrix CT improved the visualization of nodule margins, internal air-containing structures, and other fine morphologic features compared with 512 × 512 matrix reconstructions [26]. 1024 × 1024 matrix chest CT has been also reported that it significantly enhanced the visibility of interstitial lung disease findings and overall image quality compared with standard reconstruction, but it did not improve confidence in pulmonary nodule detection or attenuation classification [27].

In both the subjective and objective evaluations, image noise was significantly higher in the following order: cC > cB > cA images. These results may have been due to the thinness of the slices and smaller pixel size. Spatial resolution and image noise can both influence overall image quality, and there is a trade-off between them. The influence of these factors on the depiction of normal structures and pulmonary lesions may vary depending on the target structure. For example, according to the subjective evaluation cC images were not always effective at depicting ground-glass opacities. For consolidations, reticular opacities, and honeycombing, image quality differed significantly in the following order: cC > cB > cA images, which showed the theoretical order of high-resolution images and suggested that post-processing reconstruction of 1024 × 1024 matrix images is useful for evaluating interstitial pneumonia and bone (the bone cortex and bone trabeculae) without FOV limitations. On the other hand, for emphysema and ground-glass opacities, image quality did not necessarily follow the same pattern; i.e., cC > cB > cA, due to the increased noise associated with higher resolutions. In particular, it was considered that changing the slice thickness from 1 mm to 0.5 mm had more influence than increasing the matrix size. In this context, emerging technologies may help overcome the noise-related limitations of high-resolution imaging. Deep learning–based reconstruction is a newly developed technique for noise reduction and improvement of the signal-to-noise ratio, which may help overcome this limitation [28]. In addition, photon-counting CT has been reported to provide superior image quality compared with conventional energy-integrating detector CT for evaluating lung structures and abnormalities, owing to its improved spatial resolution and noise characteristics [29].

The main limitations of this study were as follows. First, only the image quality of normal structures and lesions was evaluated, and the value of the images for diagnosing such lesions was not adequately evaluated. A further study investigating the clinical utility of these images should be performed. Second, relatively small sample size and limited number of readers may restrict the generalizability of the findings. However, we also conducted an objective evaluation using a phantom study.

In conclusion, high-resolution CT images that were reconstructed with a 1024 × 1024 matrix using the precision matrix had equivalent spatial resolution to retargeted zoomed images reconstructed with a 512 × 512 matrix. This new post-processing 1024 × 1024 matrix reconstruction technique can provide high-resolution CT images without any FOV restrictions and effort of reconstructing retargeted zoomed image.

## Supplementary Information

Below is the link to the electronic supplementary material.

Supplementary Material 1

## Data Availability

The data that support the findings of this study are not openly available due to sensitivity.
