# Supplementary material for: High-resolution chest CT using 1024-matrix reconstruction: phantom and clinical evaluation of image quality and post-processing capability
Source: Jpn J Radiol. 2026 Mar 6;44(7):1159–67. doi: 10.1007/s11604-026-01964-0 (PMC13315464; doi:10.1007/s11604-026-01964-0)
Supplement: Supplementary file 1 — Supplementary Material 1 [file 11604_2026_1964_MOESM1_ESM.docx]

**High-Resolution Chest CT Using 1024-Matrix Reconstruction: Phantom and Clinical Evaluation of Image Quality and Post-Processing Capability**

*Supplement material*

**Materials and Methods**

*Subjects*

For the phantom study, we used the CTP528 module of the Catphan504 (The Phantom Laboratory, NY, USA) as a water phantom of 200 mm in diameter, in which an acrylic cylinder measuring 50 × 50 mm was placed, as shown in Supplement Figure S1.

*CT image acquisition and reconstruction*

For the phantom study, a 192-slice dual-source CT scanner (Somatom Force, Siemens Healthineers, Erlangen Germany) was used with following settings: collimation, 0.6 mm × 192 rows; tube voltage, 120 kVp; quality reference mAs, 150; gantry rotation time, 0.5 s; volumetric CT dose index (CTDIvol), 10.0 mGy. The phantom was set at a 70-mm offset from center of the scanner gantry. The original data obtained during the scan of the phantom were then reconstructed to produce 4 types of images. Axial CT images were reconstructed using the Bl57 kernel and an iterative reconstruction of advanced modeled iterative reconstruction (ADMIRE) strength of 3 for the lung setting. The FOV measured 300 mm in diameter. Retargeted zoomed images were also reconstructed with a FOV of half of this size; i.e., the zoomed FOV measured 150 mm in diameter. In addition, 1024 × 1024 matrix images were obtained by the post-processing reconstruction method (Precision Matrix). The 4 types of reconstructed images were produced using the following settings: pA) the lung setting with a 512 × 512 matrix and 1-mm slice thickness and interval values, pB) the lung setting with a 1024 × 1024 and 1-mm slice thickness and interval values, pC) the lung setting with a 1024 × 1024 matrix and 0.5-mm slice thickness and interval values, and pD) the retargeted zoomed lung setting with a 512 × 512 matrix and 1-mm slice thickness and interval values (Figure 1).

*Image analyses*

Phantom study

The resolution and noise characteristics of the CT images were evaluated using the task transfer function (TTF) [15-17] and noise power spectrum (NPS) [18, 19], respectively. The images used for determining the TTF were averaged from 25 mm of acrylic cylinder images in the z-axis direction to create a composite image with reduced noise. The composite edge spread function (ESF) was obtained by the circular edge method [15, 16, 20, 21], which generates a profile radially from the center of the target object using a composite image. The ESF was differentiated to generate a line spread function, and the TTF was obtained by Fourier transformation. Noise characteristics were measured from a homogenous image of the phantom, and the NPS was analyzed using the radial frequency method [16,19,22]. CT images of 40 mm in the z-axis direction were analyzed using a region of interest (ROI) with a matrix size of 128 × 128 pixels. We also obtained the attenuation profile of each CT image using a 5-line pairs/cm object of the CTP528, which is a high-contrast module of Catphan 504, and measured the curves using the ImageJ (version 1.53m) software (NIH, Bethesda, MD). The profile contrast was obtained by calculating the mean differences between the peaks and troughs of the profile for each image type.


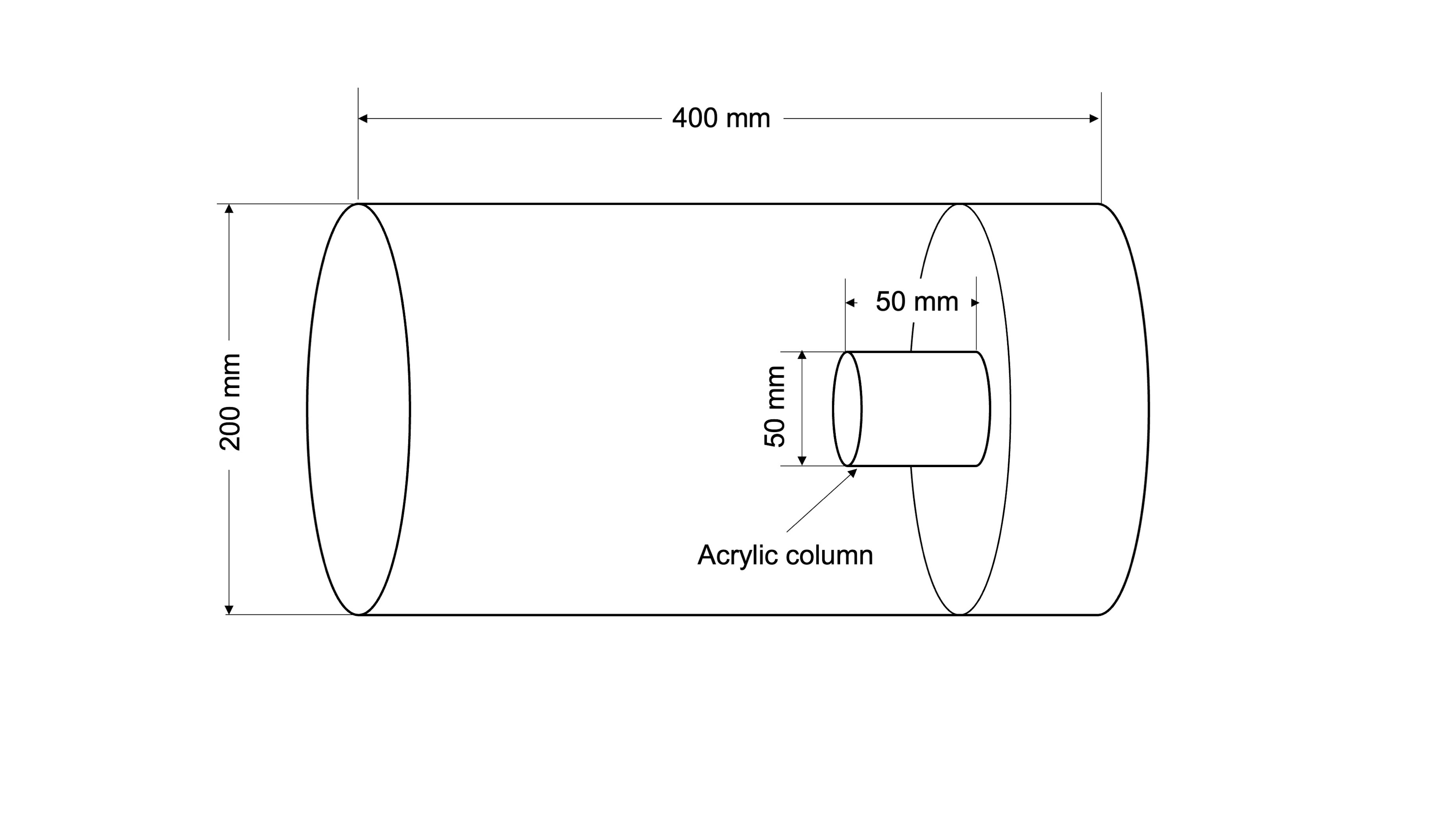


Figure S1. Water phantom overview

Columnar acrylic objects are enclosed in a cylindrical acrylic case (diameter: 200 mm) filled with water.


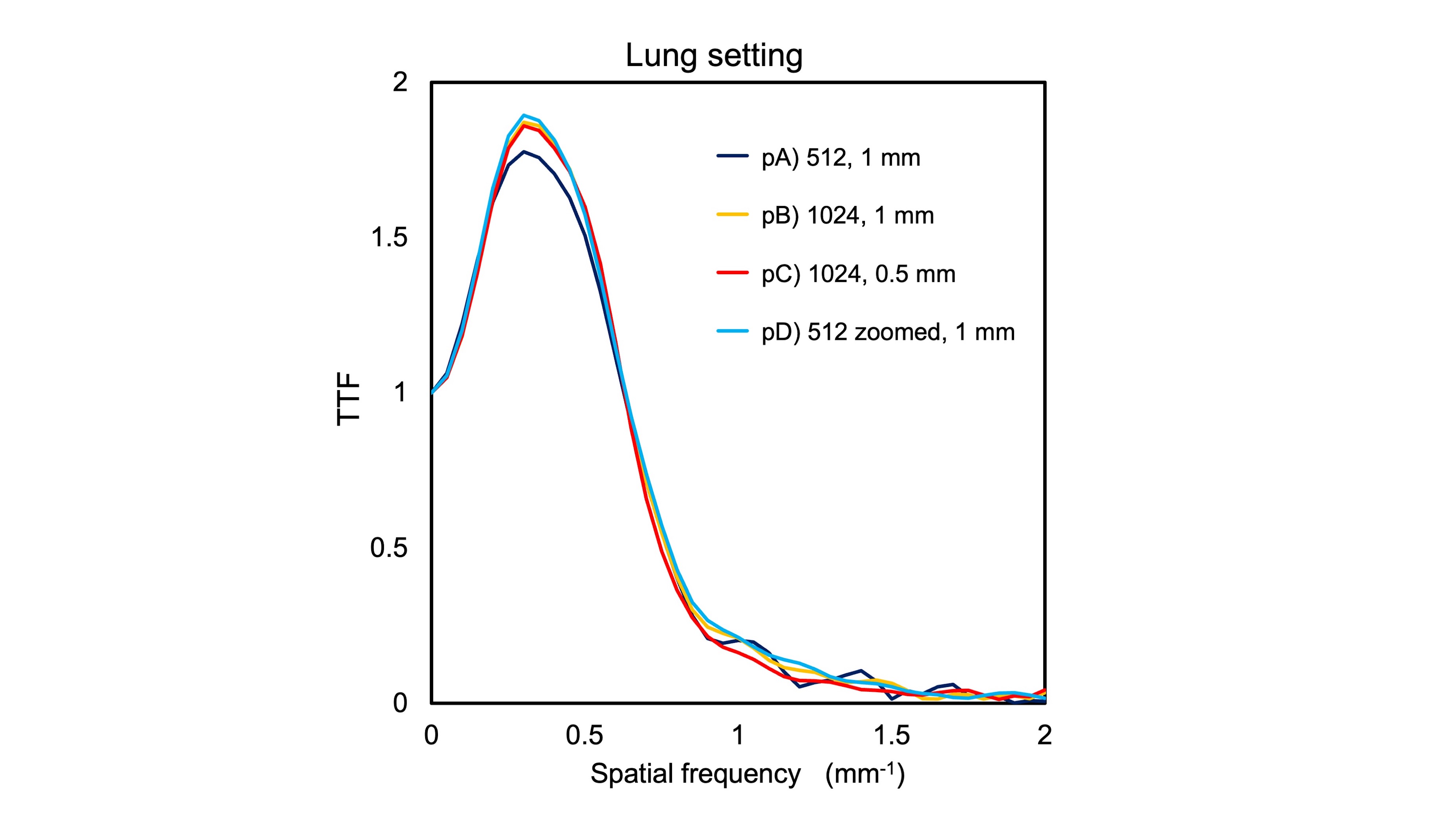


Figure S2. In-plane TTF results for CT of an acrylic column using different reconstruction modes and the lung setting

The four types of reconstructed images are obtained with the following settings: pA) the lung setting with a 512 × 512 matrix and 1-mm slice thickness and interval values, pB) the lung setting with a 1024 × 1024 matrix and 1-mm slice thickness and interval values, pC) the lung setting with a 1024 × 1024 matrix and 0.5-mm slice thickness and interval values, and pD) the retargeted zoomed lung setting with a 512 × 512 matrix and 1-mm slice thickness and interval values. In the lung setting, the peaks of the TTF curves for all image types are observed at a spatial frequency of 0.3 mm^-1^, and the mean TTF values of the pA), pB), pC), and pD) images are1.78, 1.87, 1.86, and 1.89, respectively.


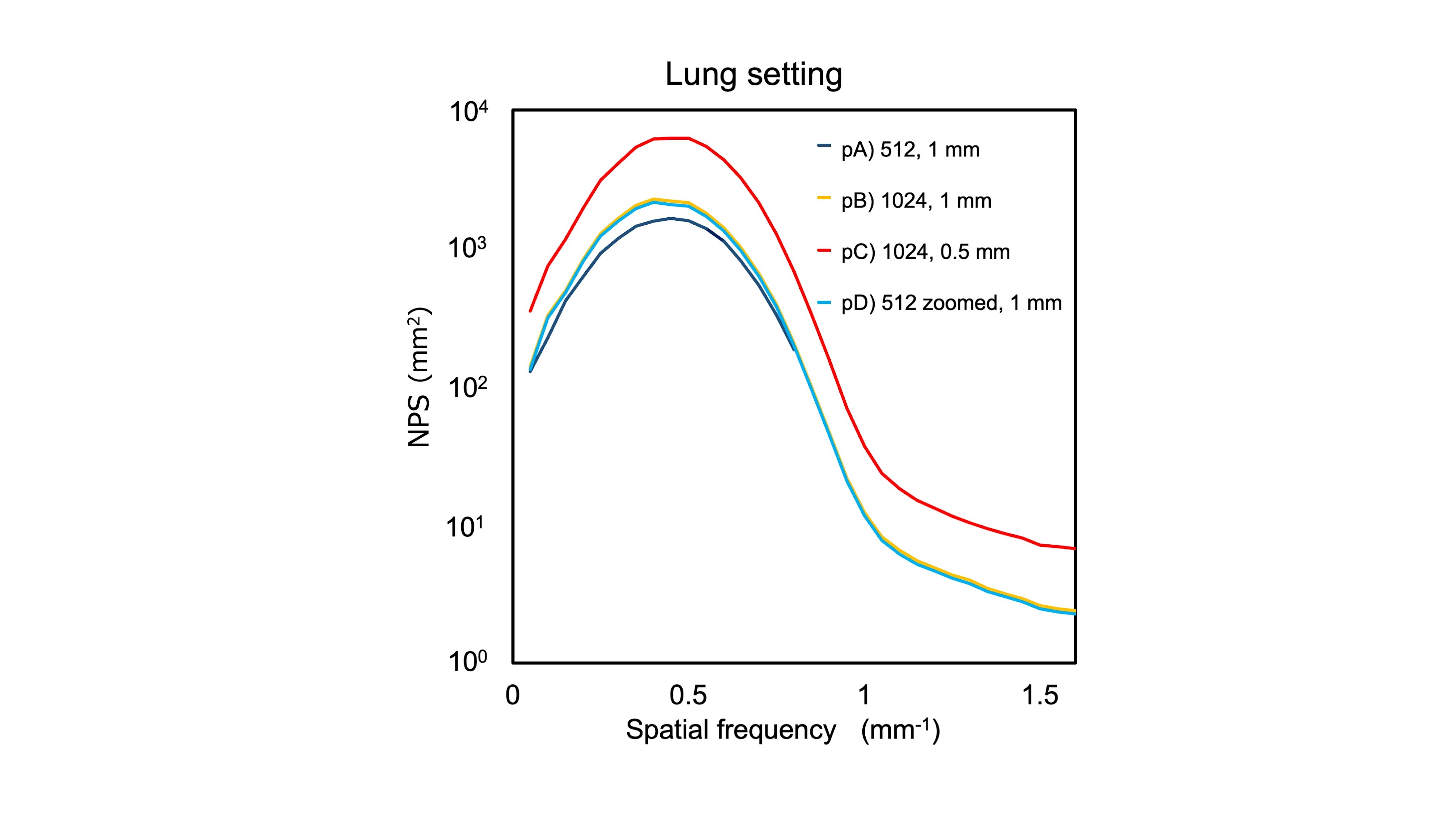


Figure S3. NPS of different reconstruction modes with the lung setting

The CT images with a 1-mm slice thickness (the pB and pD images) show similar noise characteristics, whereas the pA) images also demonstrates comparable noise characteristics, with approximately a 25% improvement at 0.5 mm^-1^. In contrast, The CT images produced with a 1024 matrix and 0.5-mm slice thickness (the pC) images) results in approximately three times the degradation in granularity at 0.5 mm^-1^ compared to pB) and pD) images.


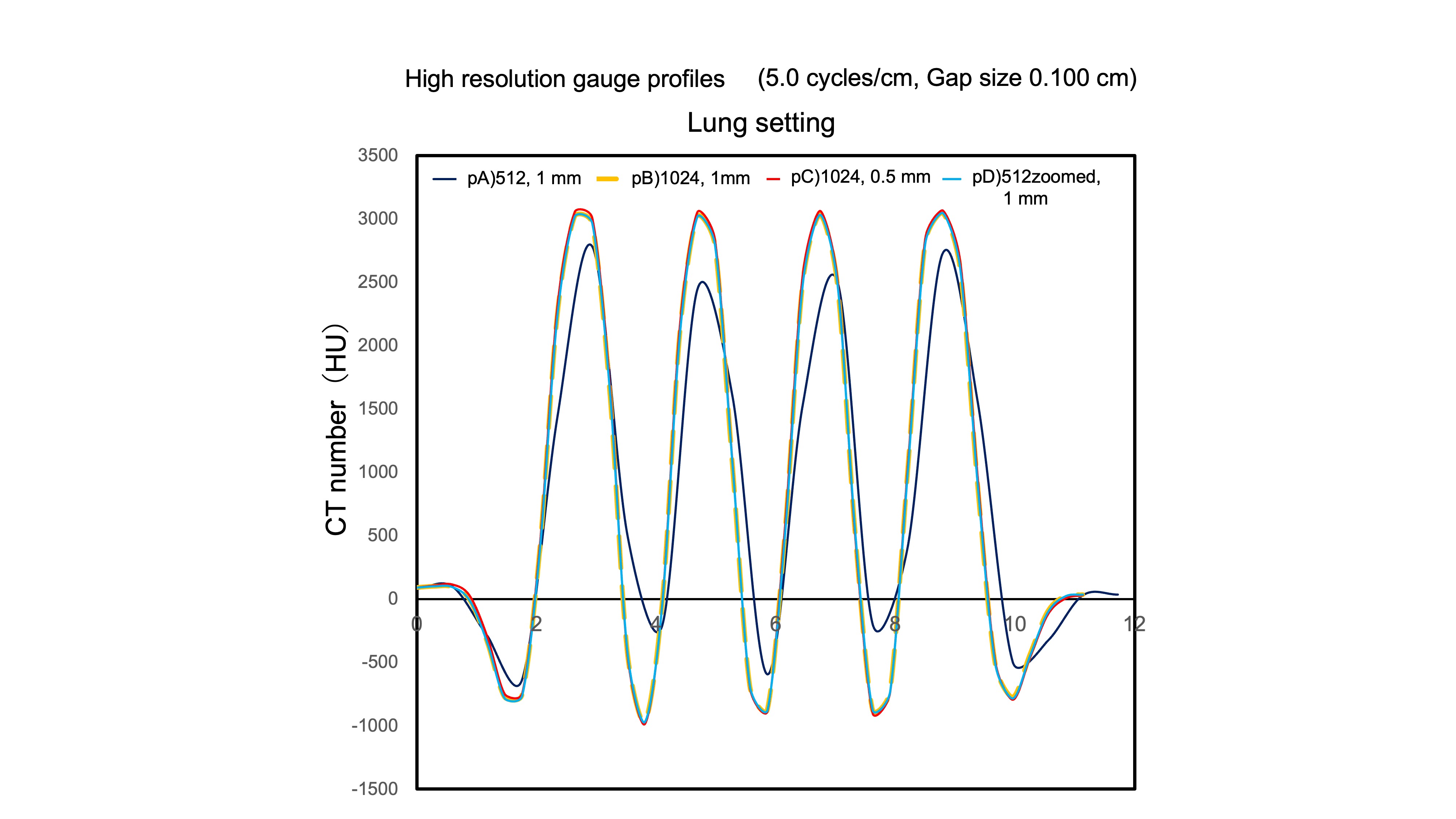


Figure S4. Profiles obtained with a 5-line pairs/cm high-contrast spatial resolution module and the lung setting

The shape of the profile for the pA) images is distorted, and its peaks are lower than those for the other image types. The mean image contrast values of the pB), pC), and pD) images are 1.3, 1.4, and 1.3 times as high as that of the pA) images, respectively. The graph shows that the lines for pB)–pD) are almost identical.
